# Segmentation of Lung Nodules on CT Images Using a Nested Three-Dimensional Fully Connected Convolutional Network

**DOI:** 10.3389/frai.2022.782225

**Published:** 2022-02-17

**Authors:** Shoji Kido, Shunske Kidera, Yasushi Hirano, Shingo Mabu, Tohru Kamiya, Nobuyuki Tanaka, Yuki Suzuki, Masahiro Yanagawa, Noriyuki Tomiyama

**Affiliations:** ^1^Department of Artificial Intelligence Diagnostic Radiology, Osaka University Graduate School of Medicine, Suita, Japan; ^2^Graduate School of Sciences and Technology for Innovation, Yamaguchi University, Ube, Japan; ^3^Medical Informatics and Decision Sciences, Yamaguchi University Hospital, Ube, Japan; ^4^Department of Mechanical and Control Engineering, Faculty of Engineering Kyushu Institute of Technology, Kitakyushu, Japan; ^5^Department of Radiology, National Hospital Organization, Yamaguchi-Ube Medical Center, Ube, Japan; ^6^Department of Radiology, Osaka University Graduate School of Medicine, Suita, Japan

**Keywords:** lung nodule, segmentation, computer-aided diagnosis, deep learning, U-Net, SegNet, watershed, graph cut

## Abstract

In computer-aided diagnosis systems for lung cancer, segmentation of lung nodules is important for analyzing image features of lung nodules on computed tomography (CT) images and distinguishing malignant nodules from benign ones. However, it is difficult to accurately and robustly segment lung nodules attached to the chest wall or with ground-glass opacities using conventional image processing methods. Therefore, this study aimed to develop a method for robust and accurate three-dimensional (3D) segmentation of lung nodule regions using deep learning. In this study, a nested 3D fully connected convolutional network with residual unit structures was proposed, and designed a new loss function. Compared with annotated images obtained under the guidance of a radiologist, the Dice similarity coefficient (DS) and intersection over union (IoU) were 0.845 ± 0.008 and 0.738 ± 0.011, respectively, for 332 lung nodules (lung adenocarcinoma) obtained from 332 patients. On the other hand, for 3D U-Net and 3D SegNet, the DS was 0.822 ± 0.009 and 0.786 ± 0.011, respectively, and the IoU was 0.711 ± 0.011 and 0.660 ± 0.012, respectively. These results indicate that the proposed method is significantly superior to well-known deep learning models. Moreover, we compared the results obtained from the proposed method with those obtained from conventional image processing methods, watersheds, and graph cuts. The DS and IoU results for the watershed method were 0.628 ± 0.027 and 0.494 ± 0.025, respectively, and those for the graph cut method were 0.566 ± 0.025 and 0.414 ± 0.021, respectively. These results indicate that the proposed method is significantly superior to conventional image processing methods. The proposed method may be useful for accurate and robust segmentation of lung nodules to assist radiologists in the diagnosis of lung nodules such as lung adenocarcinoma on CT images.

## Introduction

Lung cancer is considered one of the most serious and morbid cancers as it is the leading cause of cancer-related deaths and the most commonly detected cancer in men (Sung et al., 2021). According to the American Cancer Society, the 5-year survival rate for patients with lung cancer is 19% (Siegel et al., 2019). If lung cancer is detected in early-stage lung nodules, the survival rate improves from 10–15% to 60–80% (Diederich et al., 2002). Early detection of lung nodules is of high importance for reducing mortality rates of patients with lung cancer, because the cure rate is very low once clinical symptoms of lung cancer appear (Wu et al., 2020).

Chest X-rays and computed tomography (CT) images are used to diagnose and detect lung cancer; however, CT images are generally more effective for diagnosing lung nodules (Sone et al., 1998). According to the National Lung Screening Trial, the mortality rate owing to lung cancer among participants between the ages of 55 and 74 years with a minimum of 30 pack-years of smoking and no more than 15 years since quitting, was reduced by 20% when using CT compared with the rate when using non-CT methods (The National Lung Screening Trial Research Team, 2011). Sone et al. (1998) reported that the detection rate of lung cancer in a low-dose CT screening was 0.48%, which was significantly higher than the detection rate of 0.03–0.05% in chest radiographs performed previously in the same area. However, owing to advances in scanner technology, CT produces a large number of images; this has been time consuming and burdensome for radiologists to detect lung nodules in such a large number of cases. In addition, a radiologist's diagnosis still relies on experience and subjective evaluation.

Computer-aided diagnosis (CAD) systems have been studied to accelerate diagnosis and detection processes and support radiologists (Doi, 2007; Gurcan et al., 2009). In quantitative CAD for lung nodules, segmentation of lung nodules is an important preprocessing step (Gu et al., 2021). CAD calculates and analyzes image features such as texture features, grayscale distribution, and lung nodule volume to assist in the differential diagnosis of lung nodules (Sluimer et al., 2006). Several methods have been proposed for the segmentation of lung nodules (Gu et al., 2021). Segmentation methods for lung nodules in lung CT images are generally classified into some groups: morphological operation-based methods, region growing-based methods, region integration-based methods, optimization methods, and machine learning-based methods, including deep learning (Lecun et al., 2015).

In morphological operation methods (Haralick et al., 1987), Kostis et al. (2003) used a morphological opening operation to eliminate blood vessels attached to lung nodules with their associated connecting components. Messay et al. (2010) used a rolling ball filter with rule-based analysis for segmentation of nodules attached to the chest wall. These methods were fast and easy to implement; however, it was difficult to set the size of the morphological operator owing to the varying nodule sizes. Diciotti et al. (2008) also reported that segmentation of non-solid nodules prove to be difficult for segmentation using morphological operations.

Region growing-based methods required seed points to be set manually, and internally added voxels to nodules set until the predefined convergence criteria were satisfied. Dehmeshki et al. (2008) proposed an algorithm that used fuzzy connectivity and a contrast-based region growing to segment nodules attached to the chest wall. Kubota et al. (2011) separated nodules from the background using the region growing method by probabilistically determining the likelihood that each voxel belongs to a nodule based on local intensity values. The problem encountered with these methods was that nodules were diverse and irregular in shape; therefore, convergence criteria were difficult to set.

The watershed method was a region integration-based method (Vincent et al., 1991), wherein a grayscale image was regarded as a geographic plane, and a region was obtained by setting a marker at the local minimum of the image grayscale value and expanding the marker to neighboring pixels. Tachibana and Kido (2006) proposed a method for separation of small pulmonary nodules on CT images, segmentation of the region using the watershed method, generation of a mass model by distance transformation, and integration of the nodule regions.

Based on the energy optimization method, several methods such as level set and graph cut have been proposed. In the level set method proposed by Chan and Vese (2001), the image was described using a level set function so that the segmented contour was minimized when it matched the boundary. Farag et al. (2013) used a level set with shape before the hypothesis. Shakir et al. (2018) used a voxel intensity-based segmentation method that incorporated an average intensity-based threshold into a level-set geodesic active contour model. Graph cut (Boykov and Kolmogorov, 2004) is a method for separating objects and background by treating the input image as a graph, and it has been used to separate organ regions on CT images. Boykov and Kolmogorov (2004) incorporated the problem into a maximum flow optimization task and segmented the lung nodules using the graph cut method. Cha et al. (2018) robustly segmented lung nodules from gated 4D CT images of the respiratory system using the graph cut method. The energy optimization method segmented isolated nodules well but often failed for nodules with complex shapes, those with ground-glass opacity (GGO), and those in contact with the chest wall.

Machine learning-based lung nodule segmentation methods have been recently proposed. In these methods, features for image recognition are defined, extracted, and classified using discriminators (Ciompi et al., 2017). Liu et al. (2019) used a residual block-based dual-path network that extracted local features and rich contextual information from lung nodules, which resulted in improved performance. However, they used a fixed volume of interest (VOI) that did not allow free exploration of the nodules, which resulted in poor performance. For lung nodule segmentation, Shakibapour et al. (2019) optimally clustered a set of feature vectors consisting of intensity and shape-related features in a given feature data space extracted from predicted nodules.

Several methods based on deep learning have been currently proposed without the design of image features (Litjens et al., 2017; Kido et al., 2020). Ronneberger et al. (2015) proposed U-Net for medical image segmentation, which is now widely used. Various improvement methods have been proposed for U-Net. Tong et al. (2018) improved the performance of U-Net in nodule segmentation by including skipped connections in the encoder and decoder paths. Amorim et al. (2019) changed the architecture of U-Net and used a patch-wise approach to investigate the presence of nodules. Usman et al. (2020) proposed a two-stage method for three-dimensional (3D) segmentation of lung nodules using the residual U-Net (He et al., 2016), which incorporates a residual structure into its architecture.

Mukherjee et al. (2017) performed deep learning based segmentation of lung nodules, which uses deep learning to find the location of the object and preserves the morphological details of the object using graph cut method. Wang et al. (2017a) proposed a multi-view convolutional neural network (CNN) for lung nodule segmentation considering axial, coronal, and sagittal views around any voxel of the nodule. The same authors (Wang et al., 2017b) also proposed a central focused CNN for lung nodule segmentation. Roy et al. (2019) presented a synergistic combination of deep learning and a level set for the segmentation of lung nodules. Liu et al. (2018) used the fine-tuned the Mask R-CNN model (He et al., 2017), an object detection neural network trained on the COCO dataset (Lin et al., 2014) in order to segment lung nodules, and then tested the model on the LIDC-IDRI dataset (Armato et al., 2011).

Although many segmentation methods have been proposed for lung nodules as described above, segmentation of lung nodules with high accuracy is still difficult. For example, it is difficult to obtain a robust segmentation result when the lung nodule has GGO or is in contact with the chest wall. Therefore, in this study, a nested 3D fully connected convolutional network (FCN) using residual units (He et al., 2016) for the 3D segmentation of lung nodule regions was proposed. FCNs are the de facto standard for image segmentation, just as CNNs are the de facto standard for classification. FCNs provide robust and accurate segmentation of medical images compared to conventional methods, and new methods are being proposed one after another to improve the accuracy and robustness. While there are many applications of FCN for 2D image segmentation, most of the applications of FCN for 3D images are based on volume data obtained from CT or MRI images. In addition to diagnostic imaging, FCN has been used for segmentation of the anatomical structures of thorax and abdominal organs. In addition to diagnostic imaging, FCN has been used for segmentation of thoracic and abdominal organs to analyze their anatomical structures. The proposed model was compared with well-known deep learning models, namely 3D U-Net and 3D SegNet (Badrinarayanan et al., 2017), and the conventional image processing methods, watershed, and graph cut.

## Materials and Methods

### Study Data

CT images of 330 consecutive patients with 330 lung adenocarcinomas who had undergone surgery between 2006 and 2014 at the Saiseikai Yamaguchi General Hospital (185 men and 168 women; mean age: 69.7 ± 9.7 years; range: 30–93 years) were used. CT images were acquired using Somatom Definition and Somatom Sensation 64 (Siemens, Erlangen, Germany) and were obtained at the suspended end-inspiratory effort in the supine position without intravenous contrast material. The acquisition parameters were as follows: collimation, 0.6 mm; pitch, 0.9; rotation time, 0.33 s/rotation; tube voltage, 120 kVp; tube current, 200 mA; and field of view, 200 or 300 mm. All image data were reconstructed with a high spatial frequency algorithm and reconstruction thickness, and the intervals were 1.0 and 2.0 mm for 141 and 212 patients, respectively. The annotation of all lung nodules for evaluation was performed under the guidance of a board-certified radiologist.

### Datasets

For 3D CT images containing 330 lung nodules, the lung nodule regions were cropped to size of 128^*^128^*^64 and these images were divided into five parts. For four of them, data augmentation (rotation processing of 15° each in the x-y slice plane, and mirroring processing for the x-, y-, and z-axes) was performed. 96 lung nodule images were generated from one lung nodule image, and used as the training data set. The remaining one was used as a test dataset. This process was performed five times while changing the test dataset (5-fold cross validation).

### Network Architecture of the Proposed Model

**Figure 1:**
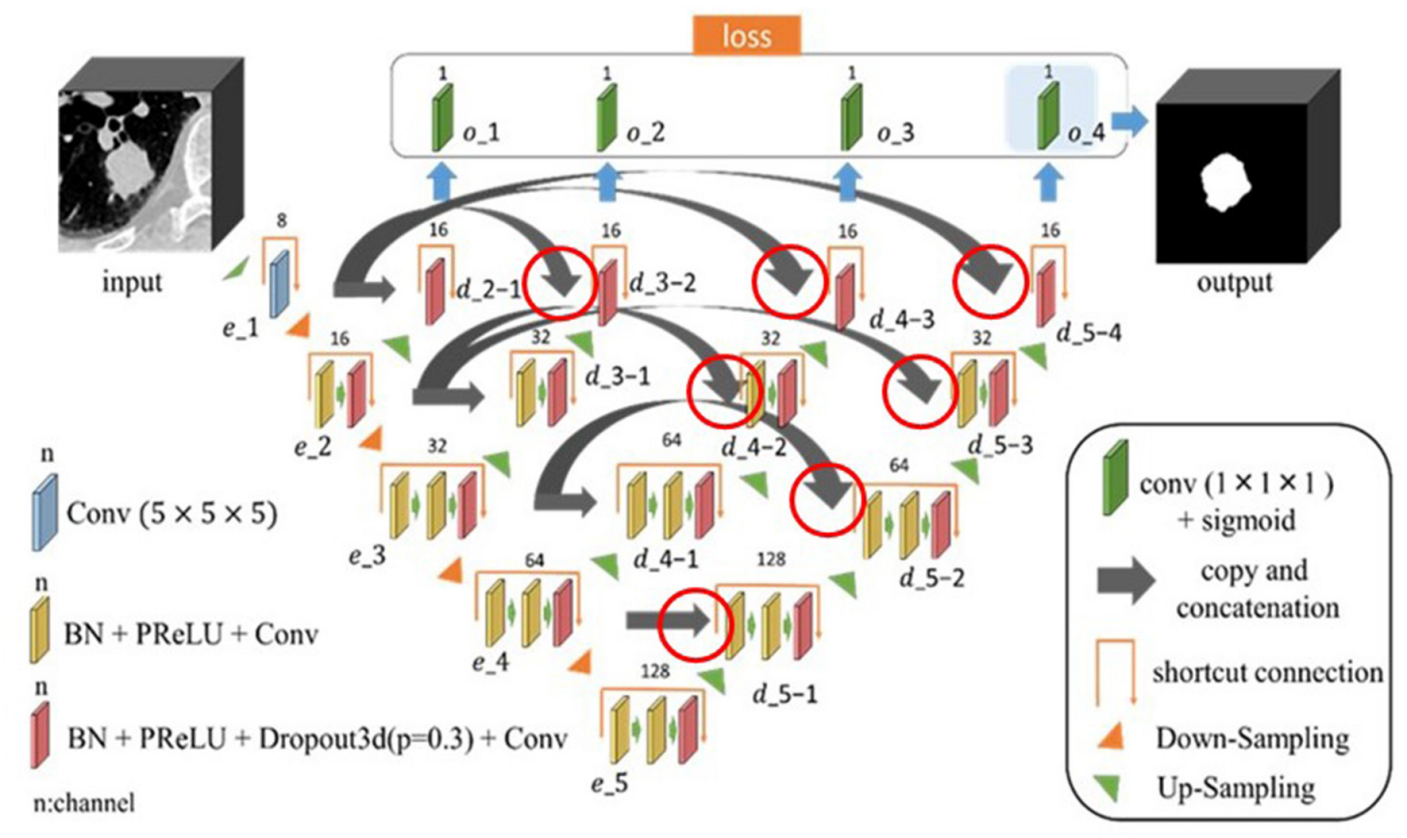
Architecture of the proposed nested three-dimensional (3D) fully connected convolutional network. The connections are indicated by the red circles, where the encoder and decoder are connected by concatenation.

The proposed model is shown in Figure 1. Image features were extracted from the input 3D CT image using a single encoder network, and image features output from each block (e_2 to e_5) of the encoder network were used to create the region segmentation result (region map). The decoder network connected to each block (e_2 to e_5) was also used to create the region map. The created region map (output of o_1 to o_4) was substituted into the loss function to calculate the loss value. The outputs included the deepest encoder network (e_1 to e_5), deepest decoder network (d_5-1 to d_5-4), and region map created by o_4. Furthermore, each block had a housing unit structure, and all blocks except e_1 incorporated a dropout layer (Dropout3d) before the final convolutional layer. In the proposed model, encoder and decoder are connected by concatenation. The hyperparameter tuning of the model was done experimentally.

### Loss Function

The loss function used in this study is given in Equation 1.


(1) $$\begin{array}{l} loss\left(x.y\right)=\lambda loss_{b}\left(x,y\right)+\left(1.0-\lambda \right)loss_{d}\left(x,y\right),\lambda \epsilon \left[0.0,1.0\right] \end{array}$$

In this equation, *loss*_*b*_(*x, y*) is the binary cross entropy, *loss*_*d*_(*x, y*) is the Dice loss, *x* is the predicted image, and *y* is the annotated image. The binary cross entropy and Dice loss terms in the equation of the loss function are multiplied by coefficients λ and (1.0-λ), which range from 0.0–1.0. The tree-structured Parzen Estimator was used to determine hyperparameters (Ozaki et al., 2020). Therefore, Optuna, a hyperparameter auto-optimization framework for machine learning was used (Akiba et al., 2019).

### Residual Unit

**Figure 2:**
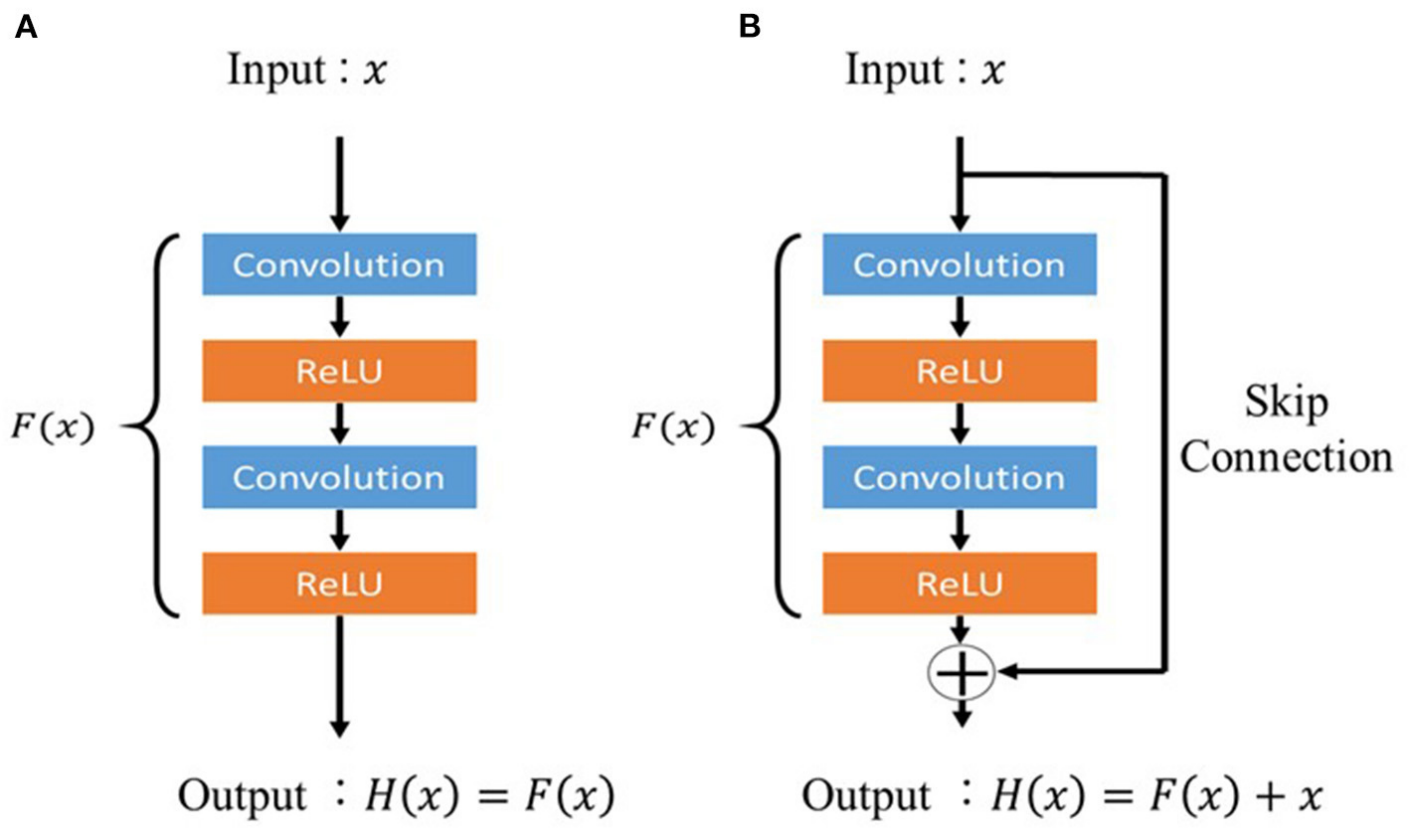
Architecture of the residual unit. **(A)** Conventional feed-forward neural network and **(B)** residual unit.

Residual unit is a technique for deepening the CNN used in the residual network (ResNet) proposed by He et al. (2016). Deepening the layers of CNNs usually enables more advanced and complex feature extraction; however, simply deepening the layers of CNNs can deteriorate the performance owing to problems such as gradient vanishing. Activation functions such as ReLU and dropout have been proposed as a solution to this problem; however, training does not proceed when the CNN layers are made deeper than a certain level even when these functions are applied. Therefore, a deep residual learning framework (residual unit) is devised. In a conventional CNN, if the input is *x* and the output is *H*(*x*), the network will appear, as shown in Figure 2A. In contrast, the residual unit has a skip connection structure where the input does not pass through the convolutional layer, as shown in Figure 2B, and is trained using Equation 2. The two convolutional layers were then trained using Equation 3, implying the presence of residuals.


(2) $$\begin{array}{l} H\left(x\right)=F\left(x\right)+x \end{array}$$

(3) $$\begin{array}{l} F\left(x\right)=H\left(x\right)-x \end{array}$$

This makes it easy to learn *F*(*x*) even when the difference between *x* and *H*(*x*) is small. In the proposed model, the residual unit structure prevents the gradient from decreasing, even in the deepest layers of the model, and the image features around the lung nodules can be properly trained.

### Comparison With Different Methods

Our proposed method was compared with the following four methods that have already been published. 3D U-Net and 3D SegNet were used as segmentation methods using deep learning. Moreover, the watershed method and graph cut method were used as conventional image processing methods.

### U-Net

U-Net is an object segmentation model proposed by Ronneberger et al. (2015) for biomedical images and is currently the best known segmentation method for medical images. U-Net is an FCN (Shelhamer et al., 2017), and the difference between U-Net and FCN is that the information used for coding is also used for decoding the convolutional image. In this study, the mini-batch gradient descent method was used to optimize the parameters of the network model. Five-fold cross-validation was performed.

### SegNet

SegNet is a segmentation model for object regions proposed by Badrinarayanan et al. (2017) in 2016. SegNet has the same encoder and decoder structure as U-Net. However, while U-Net uses convolution transpose, SegNet uses unpooling. In addition, unlike U-Net, SegNet does not have a skip connection structure. In this study, the mini-batch gradient descent method was used to optimize the parameters of the network model. Adam was used as an algorithm to update the parameters (Kingma and Ba, 2015). Cross entropy was used for loss function, and 5-fold cross-validation was performed.

### Watershed Method

The segmentation of lung nodules using the watershed method comprises two main steps. The first step is to segment the rough region of the lung nodule by determining a threshold value to separate the lung nodule region from the rest of the lung. The rough region is the area that includes the blood vessels and trachea adjacent to the nodule after removing the chest wall and other parts adjacent to the nodule. In the second step, a model of the lung nodule region was created using distance transformation. The lung nodule region and blood vessel region were segmented based on gray scale information, and the lung nodule region was segmented. The VOI was set to 128 × 128 × 64 voxels, similar to the proposed method, and the center of the VOI was set to the center of the lung nodule.

### Graph Cut Method

The graph cut method has the following features: it can reflect the likelihood and boundedness inside the region in a well-balanced manner, globally optimize the energy, and can be easily extended to multidimensional data. For objects with a known shape, the segmentation accuracy can be further improved by setting an appropriate shape energy. In general, the energy is given in the form of a linear sum of the region term *region*(*L*) and boundary term *boundary*(*L*), as shown in Equation 4.


(4) $$\begin{array}{l} E\left(L\right)=Region\left(L\right)+Boundary\left(L\right) \end{array}$$

The region where the energy *E*(*L*) is minimized is determined and segmented.

### Evaluation Parameters

The accuracy of the proposed method is quantitatively evaluated using performance measures such as the Dice similarity coefficient (DS) and intersection of union (IoU). These measures are calculated by determining the difference between the results of segmentation and a manually annotated reference standard. DS was calculated using Equation 5, and IoU was calculated using Equation 6. In the equations, *R* is the manually annotated reference standard and *S* is the result of segmentation.


(5) $$\begin{array}{l} DS\left(R,S\right)=\frac{2\times |R\cap S|}{\left|R|+|S\right|} \end{array}$$

(6) $$\begin{array}{l} IoU\left(R,S\right)=\frac{\left|R\cap S\right|}{\left|R\cup S\right|} \end{array}$$

### Computation Environment

The proposed model and four different methods were implemented on a custom-made Linux-based computing server equipped with GeForce GTX 1080 Ti (NVIDIA Corporation, Santa Clara, CA, USA) and Xeon CPU E5-2623 v4 (Intel Corporation, Santa Clara, CA, USA). The deep learning model was implemented using PyTorch. In addition, a hyperparameter search was performed using an open-source hyperparameter auto-optimization framework, Optuna (Preferred Networks, Inc., Tokyo, Japan).

## Results

Table 1 shows a comparison between the proposed method and other four methods. The results of the proposed method were 0.845 ± 0.008 and 0.738 ± 0.011 for DS and IoU, respectively. The DS and IoU were 0.822 ± 0.009 and 0.711 ± 0.011, respectively, for 3D U-Net and 0.786 ± 0.011 and 0.660 ± 0.012, respectively, for 3D SegNet. Therefore, the proposed method was significantly better than 3D U-Net and 3D SegNet. Moreover, the results of the watershed method were 0.628 ± 0.027 and 0.494 ± 0.025 for DS and IoU, respectively, and the results of the graph cut method were 0.566 ± 0.025 and 0.414 ± 0.021, respectively. The proposed method was also significantly better than the watershed and graph cut methods.

**Table 1:** Comparison of the proposed method with four segmentation methods.

	**DS (**Mean±SD)****	**IoU (**Mean±SD)****
Proposed	0.845 ± 0.008	0.738 ± 0.011
3D U-Net	0.822 ± 0.009^*^	0.711 ± 0.011^*^
3D SegNet	0.786 ± 0.011^**^	0.660 ± 0.012^**^
Watershed	0.628 ± 0.027^**^	0.494 ± 0.025^**^
Graph cut	0.566 ± 0.025^**^	0.414 ± 0.021^**^

*Comparison with the proposed method*.

* *P < 0.01*.

** *P < 0.001*.

The average processing times per case for the proposed model, 3D U-Net, and 3D SegNet using graphics processing unit are shown in Table 2. The average segmentation time of the proposed model was 0.283 ± 0.002 s, which was longer than that of the 3D U-Net (0.160 ± 0.001 s) and 3D SegNet (0.083 ± 0.001 s). However, the percentage of the total processing time for analyzing lung nodules was short, and no practical problems were expected.

**Table 2:** Average processing time per case for the proposed model, 3D U-Net, and 3D SegNet.

	**Average processing time Mean±SD (s)**
Proposed	0.283 ± 0.002
3D U-Net	0.160 ± 0.001^***^
3D SegNet	0.083 ± 0.001^***^

*Comparison with the proposed method*.

*** *P < 0.0001*.

**Figure 3:**
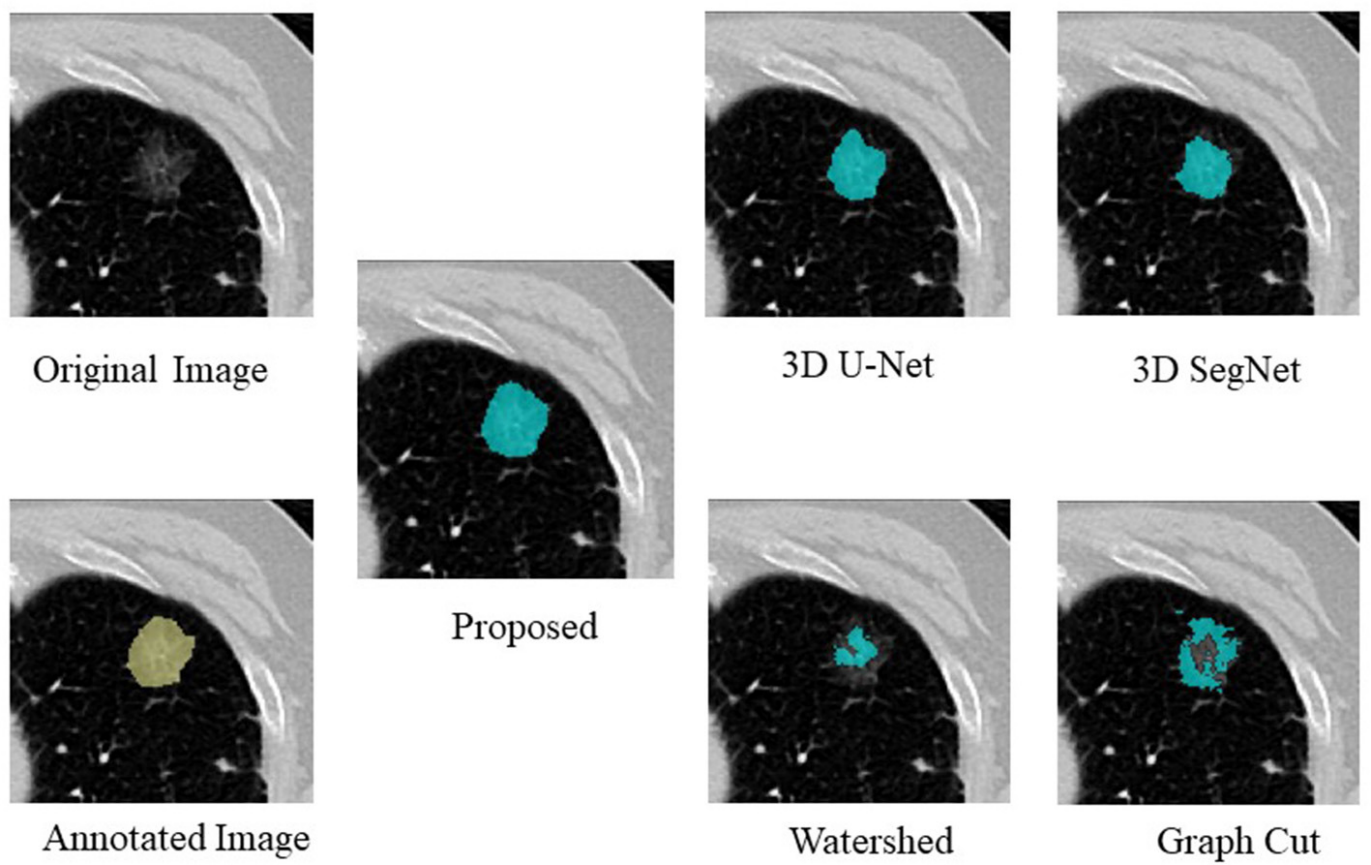
Examples of segmentation results in the case of a GGO nodule.

An example of segmentation in the case of a GGO nodule is shown in Figure 3, Table 3. The proposed method was considered the best, followed by 3D U-Net and 3D SegNet. There were instances wherein the watershed and graph cut methods failed to segment the GGO regions at the edges and inside the nodule.

**Table 3:** Comparison of the proposed method with four segmentation methods in the case of a GGO nodule.

	**DS**	**IoU**
Proposed	0.886	0.795
3D U-Net	0.849	0.738
3D SegNet	0.835	0.719
Watershed	0.310	0.184
Graph cut	0.573	0.401

**Figure 4:**
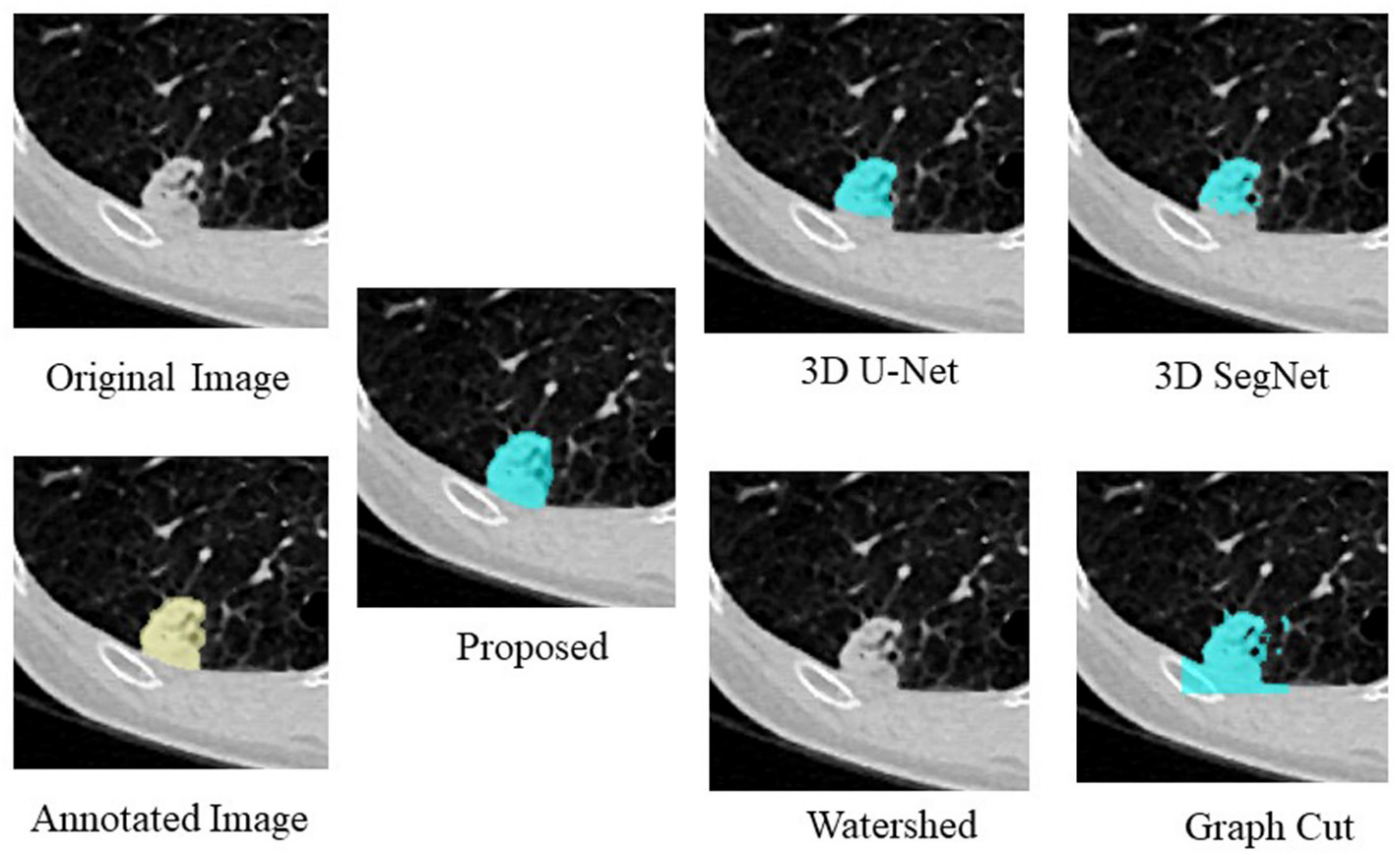
Examples of segmentation results in the case of a nodule attached to the chest wall.

Figure 4, Table 4 show an example of segmentation in the case of a nodule attached to the chest wall. 3D U-Net and 3D SegNet failed to segment the boundary region when the nodule was in the adjacent chest wall, while the graph cut method identified the chest wall region contiguous to the nodule as the nodule. The watershed method failed to segment lung nodules.

**Table 4:** Comparison of the proposed method with four segmentation methods in the case of a nodule attached to the chest wall.

	**DS**	**IoU**
Proposed	0.772	0.628
3D U-Net	0.559	0.388
3D SegNet	0.528	0.359
Watershed	0.000	0.000
Graph cut	0.504	0.336

## Discussion

In this study, a nested 3D FCN for segmenting lung nodule regions on CT images was proposed, and its segmentation accuracy was 0.845 ± 0.007 and 0.738 ± 0.011 for DS and IoU, respectively. These results were better than those obtained using other segmentation methods used in comparison experiments. The main contribution of the proposed model could segment appropriate regions for lung nodules with GGO and lung nodules attached to the chest wall, which tended not to be segmented by well-known deep learning models, namely 3D U-Net and 3D SegNet, and by the conventional image processing methods, watershed, and graph cut. This is because the region map was created using the decoder network connected to e_2 to e_5 of the encoder network part of the proposed model, and the loss was calculated and trained using this decoder network. Therefore, it can be inferred that even the shallow parts of the encoder network in the model (e_1 and e_2) are trained to segment appropriate lung nodule image features and that the encoder network in the deep part of the model (e_3 onward) increased the number of feature patterns to be segmented and could segment more advanced features. In addition, the residual unit structure adopted in the proposed model prevents the gradient from disappearing in the deeper layers of the model and allows the model to learn the features of the complex and faint edges of lung nodules, which improves segmentation accuracy and allows the model to accurately segment lung nodules with GGO and nodules attached to the chest wall.

**Figure 5:**
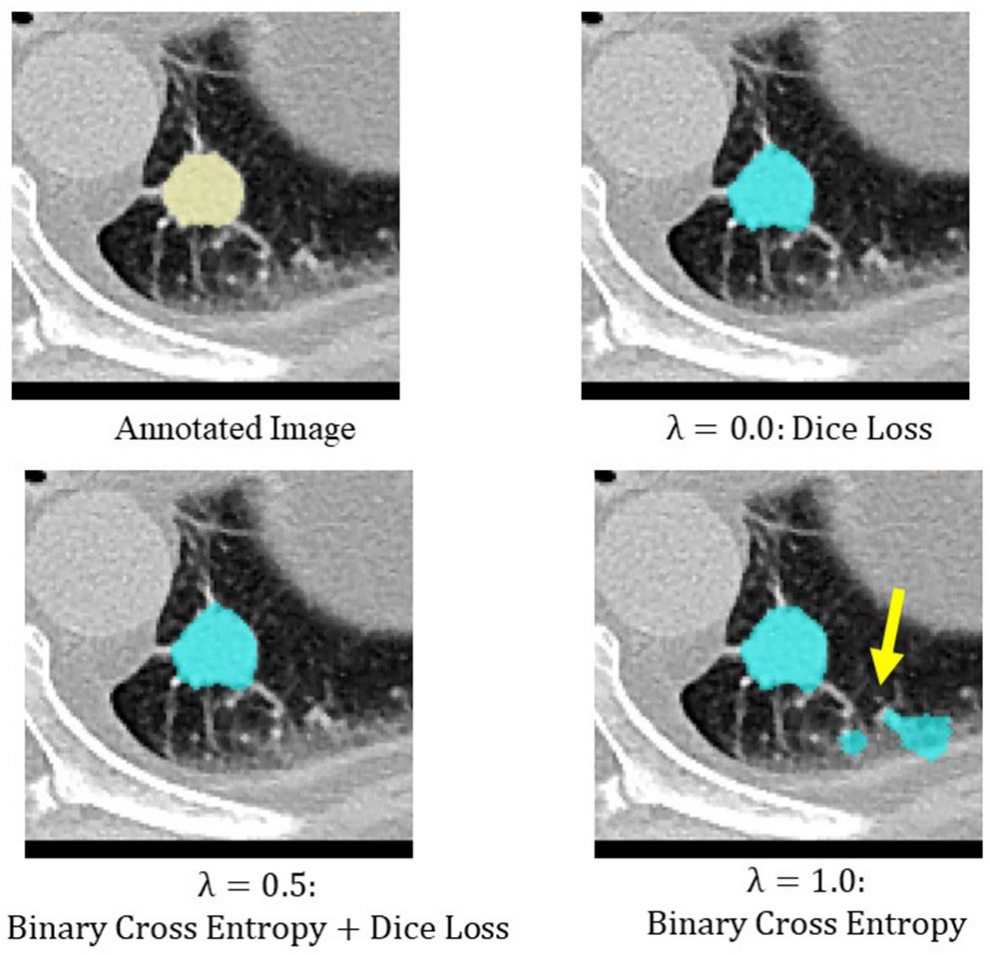
An example of extraction results when the value of λ was changed. Under-extraction was observed when only Dice loss was used as the loss function (λ = 0.0).

**Figure 6:**
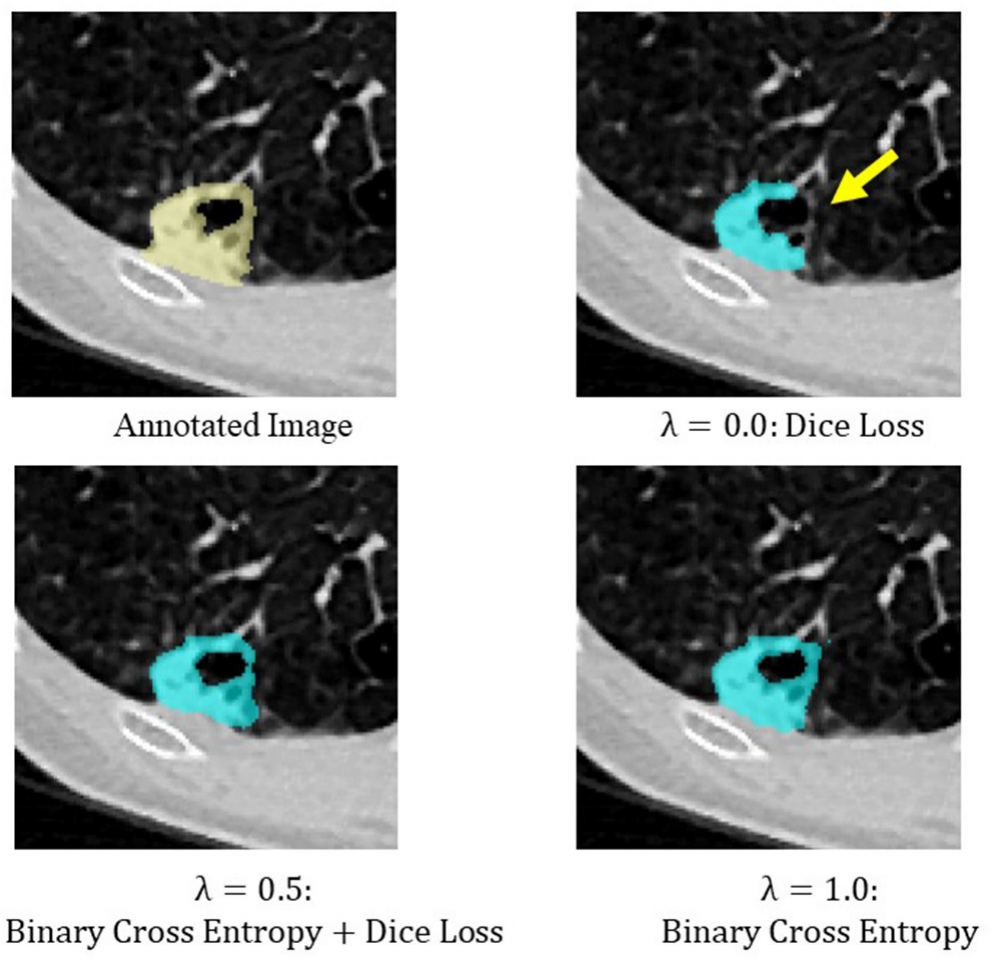
An example of extraction results when the value of λ was changed. Over-extraction was observed when only binary cross entropy was used as the loss function (λ = 1.0).

Binary cross entropy calculates the loss for each pixel value of the prediction result and the annotated image, while Dice loss calculates the loss by calculating the coincidence between the prediction result and the region of the annotated image. In general, when the object to be extracted is too small for the background, Dice loss is more sensitive than binary cross entropy, but when the shape of the object to be extracted is complex, binary cross entropy is more sensitive (Bertels et al., 2019; Zhu et al., 2019). Also, compared to binary cross entropy, Dice loss is prone to unstable learning. For this reason, the optimal coefficients for combining both were determined by Bayesian optimization. The cases of binary cross entropy or Dice loss as the only loss function were experimentally determined (Figures 5, 6). In these cases, under- or over-extraction were observed in the extracted images. However, by optimizing λ, good extraction results were obtained.

Regarding the average segmentation time for nodules, the proposed model took 0.283 ± 0.002 s, which is longer than that required by the other deep learning models. However, if the segmentation of nodules is considered as a preprocessing step for CAD, there is no practical problem.

A limitation of this study was that the number of nodules used was relatively small, and all nodules were adenocarcinomas. This is because CT images collected for this study were from cases that were indicated for surgery for lung adenocarcinoma. Therefore, more cases other than lung adenocarcinoma need to be collected to assess various nodule morphologies.

In conclusion, the effectiveness of our proposed lung nodule segmentation method was verified by comparison with other nodule segmentation methods. The proposed method provides an effective tool for CAD of lung cancer, where accurate and robust segmentation of lung nodules is important. This tool may also enhance the differential diagnosis of lung nodules, which is currently performed manually. In the future, improvement of the accuracy of segmentation for all types of lung nodules is planned.

## Data Availability Statement

The raw data supporting the conclusions of this article will be made available by the authors, without undue reservation.

## Ethics Statement

The studies involving human participants were reviewed and approved by Saiseikai Yamaguchi General Hospital. Written informed consent for participation was not required for this study in accordance with the national legislation and the institutional requirements.

## Author Contributions

SKido, SKide, YH, and SM contributed to the conception and design of this study. NTa organized the database. SKido and SKide created the model and performed the experiments. SKido, YH, SM, and TK performed model evaluation and statistical analysis. SKido wrote the first draft of the manuscript. MY and NTo contributed to the clinical evaluation. All authors contributed to the revision of the manuscript and read and approved the submitted version.

## Funding

This work was supported by JSPS KAKENHI Grant Number 21H03840.

## Conflict of Interest

The authors declare that the research was conducted in the absence of any commercial or financial relationships that could be construed as a potential conflict of interest.

## Publisher's Note

All claims expressed in this article are solely those of the authors and do not necessarily represent those of their affiliated organizations, or those of the publisher, the editors and the reviewers. Any product that may be evaluated in this article, or claim that may be made by its manufacturer, is not guaranteed or endorsed by the publisher.
